# Case Report: High Doses of Intravenous Immunoglobulins as a Successful Treatment for Late Onset Immune Agranulocytosis After Rituximab Plus Bendamustine

**DOI:** 10.3389/fimmu.2021.798251

**Published:** 2022-01-10

**Authors:** Ramon Diez-Feijóo, Juan Jose Rodríguez-Sevilla, Concepcion Fernández-Rodríguez, Solange Flores, Carmen Raya, Ana Ferrer, Luis Colomo, Antonio Salar

**Affiliations:** ^1^ Department of Hematology, Hospital del Mar, Barcelona, Spain; ^2^ Applied Clinical Research in Hematological Malignancies, Hospital del Mar Medical Research Institute (IMIM), Barcelona, Spain; ^3^ Banc de Sang i Teixits, Hospital del Mar, Barcelona, Spain; ^4^ Department of Pathology, Hospital del Mar, Barcelona, Spain

**Keywords:** immunoglobulin, Waldenström’s macroglobulinemia, bendamustine, agranulocytosis, rituximab

## Abstract

Late onset neutropenia (LON) related to rituximab or rituximab plus chemotherapy is defined as an unexplained absolute neutrophil count of ≤1.5 × 10^9^/L starting at least four weeks after the last rituximab administration. LON is infrequent and its pathophysiology remains unknown. There are no guidelines or consensus strategies for the optimal management of patients developing LON. The majority of the patients recover promptly with no specific treatment and only some cases need to be managed with granulocytic colony stimulating factor (G-CSF), usually with a rapid response. Here, we describe a 69-year-old patient with Waldenström’s macroglobulinemia who presented a septic event in the context of severe LON after rituximab plus bendamustine. The diagnosed of agranulocytosis was established by bone marrow examination. Interestingly, anti-neutrophil antibodies bound to the patient’s granulocytes were found suggesting an autoimmune mechanism. The patient did not respond to G-CSF but achieved a rapid response after high doses of intravenous immunoglobulins with full white blood cell recovery.

## Introduction

Late onset neutropenia (LON) induced by rituximab (R) is usually defined as an unexplained absolute neutrophil count of ≤1.5 × 10^9^/L (corresponding to neutropenia of grade 2–4 according to National Cancer Institute Common Toxicity Criteria) starting at least 4 weeks after the last treatment with R (1–3). The incidence of LON after R varies among series from 3% to 27%, although grade IV neutropenia is less common (3-11%) (4). The mechanisms behind LON are poorly defined.

There are no specific recommendations for the management of LON. Some patients may recover promptly with no specific treatment and some may need to be managed with granulocytic colony stimulating factor (G-CSF), usually with a rapid response (1–3).

We present here a rare case of late onset autoimmune agranulocytosis after an R-bendamustine (RB) regimen in a patient with Waldenström macroglobulinemia (WM) who did not respond to G-CSF and was effectively managed with high doses of intravenous immunoglobulins (IVIG).

## Case Report

A 69-year-old male was admitted to the hospital for febrile neutropenia. Twenty-three days before admission he had received the second cycle of RB as second-line treatment for WM. No new medication was started during this period of time. Treatment was initiated due to progressive thrombocytopenia and an increase of the monoclonal component. Prior to RB, he had received 8 R cycles as first-line therapy without major complications.

At the time of admission, physical examination was normal. Complete blood test revealed haemoglobin 93 g/L (120-150 g/L), reticulocytes 22 x10^9^/L (50-100 x10^9^/L), mean corpuscular volume 90 fL (80-100 fL), total white cell count 0.07 x10^9^/L (4-10 x10^9^/L), neutrophils 0.00 x10^9^/L (2-7 x10^9^/L), lymphocytes 0.06 x10^9^/L (1-3 x10^9^/L), platelets 79 x10^9^/L (150-400 x10^9^/L), lactate dehydrogenase 345 IU/L (240-480 IU/L), bilirubin 0.7 mg/dL (0.2-1.2 mg/dL), haptoglobin 80 (30-200mg/dl), total proteins 5.6 g/dL (6-8.3 g/dL) and albumin 2.8 g/dL (3.8-5.1 g/dL). Serum protein electrophoresis and immunofixation showed a monoclonal IgM kappa peak of 6.6 g/L. The immunoglobulin dosage in serum was as follows: IgG 608 mg/dL (700-1600 mg/dL), IgA 113 mg/dL (70-400 mg/dL), IgM 613 mg/dL (40-240 mg/dL). ANA antibodies were negative. A direct antiglobulin test was negative. A blood smear confirmed the cytopenias without other pathological findings. Microbiological cultures revealed a urinary infection due to *Escherichia coli* with associated bacteriemia.

Broad-spectrum antibiotic therapy and G-CSF were started. Platelets normalized once sepsis parameters were controlled. Despite adequate antibiotic coverage and blood culture negativization, the patient remained feverish for the next 10 days and with a neutrophil count of 0 x 10^9^/L. A body computerized tomography scan ruled out the presence of infection. Serologies for human immunodeficiency virus, hepatitis B virus, hepatitis C virus, Epstein-Barr virus, leishmania, cryptococcus, treponema pallidum, and parvovirus showed no acute infection. The polymerase chain reaction for cytomegalovirus and parvovirus in blood was negative.

**Figure 1:**
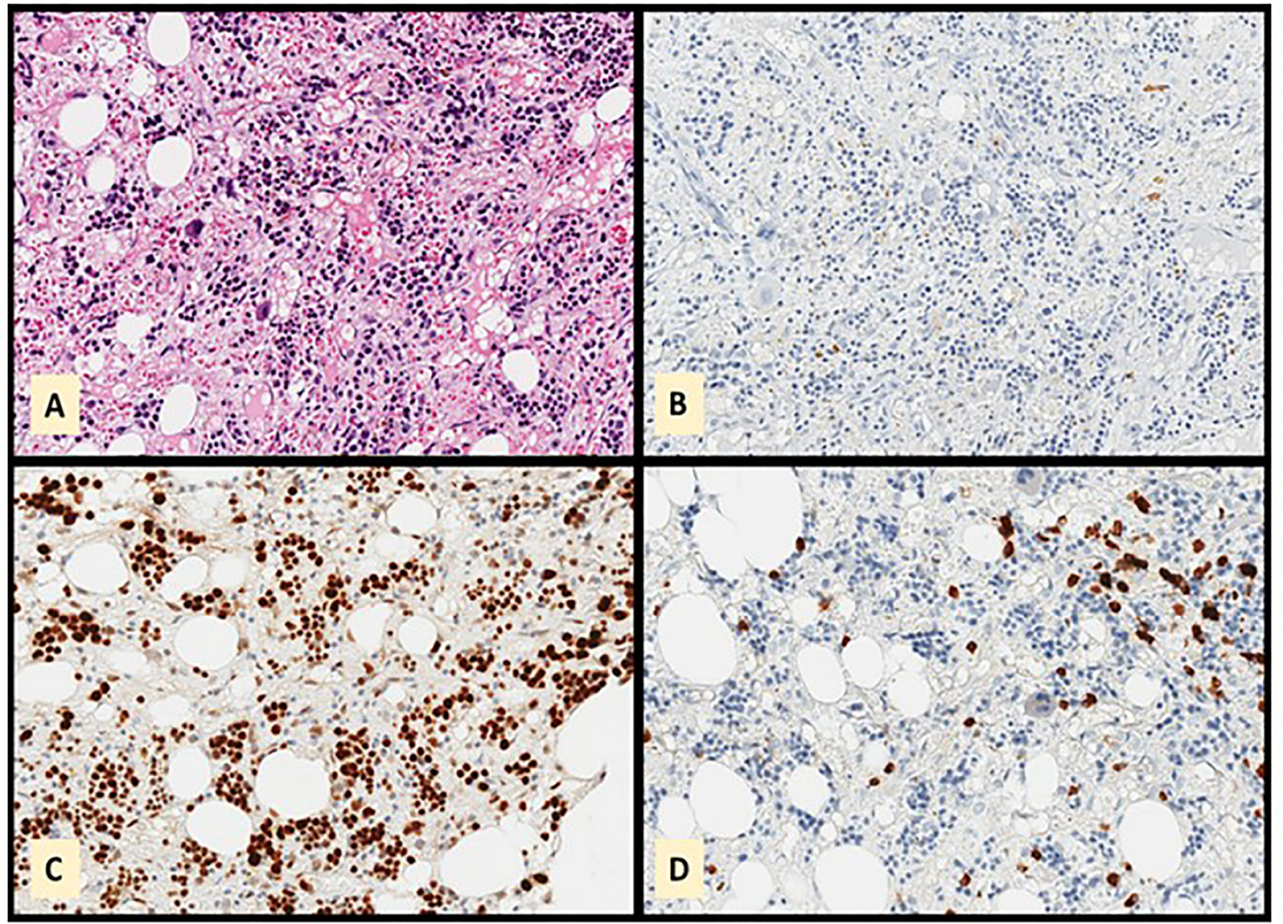
Bone marrow biopsy showing absence of granulocytic series. The cellularity observed in the Hematoxylin-Eosin stain **(A)** is myeloperoxidase negative **(B)**. LMO2 **(C)** and CD79a **(D)** showing that the observed cellularity corresponds mainly to red and plasma cells.

**Figure 2:**
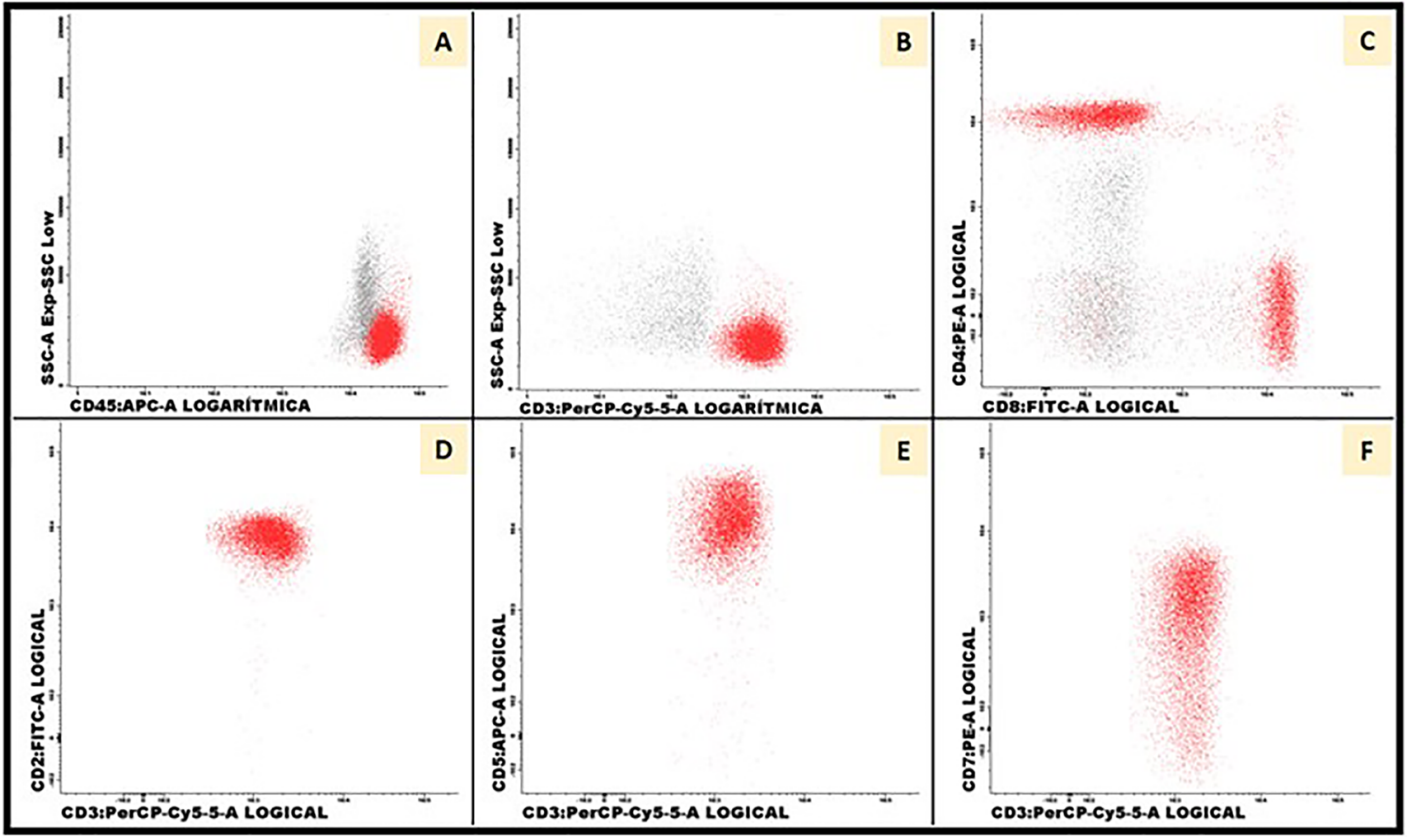
Blood immunophenotypic study of T lymphocytes. Flow cytometry was performed in peripheral blood with 50.000 total events acquired per tube (FACS Canto II, BD Biosciences). T lymphocytes were gated using CD3 antigen **(A, B)**. Distribution of CD4+ (57%) and CD8+ (38%) populations were normal **(C)**. Expression of PAN-T antigens CD2 (100%), CD5 (100%) and CD7 (90%) were normal **(D–F)**.

**Figure 3:**
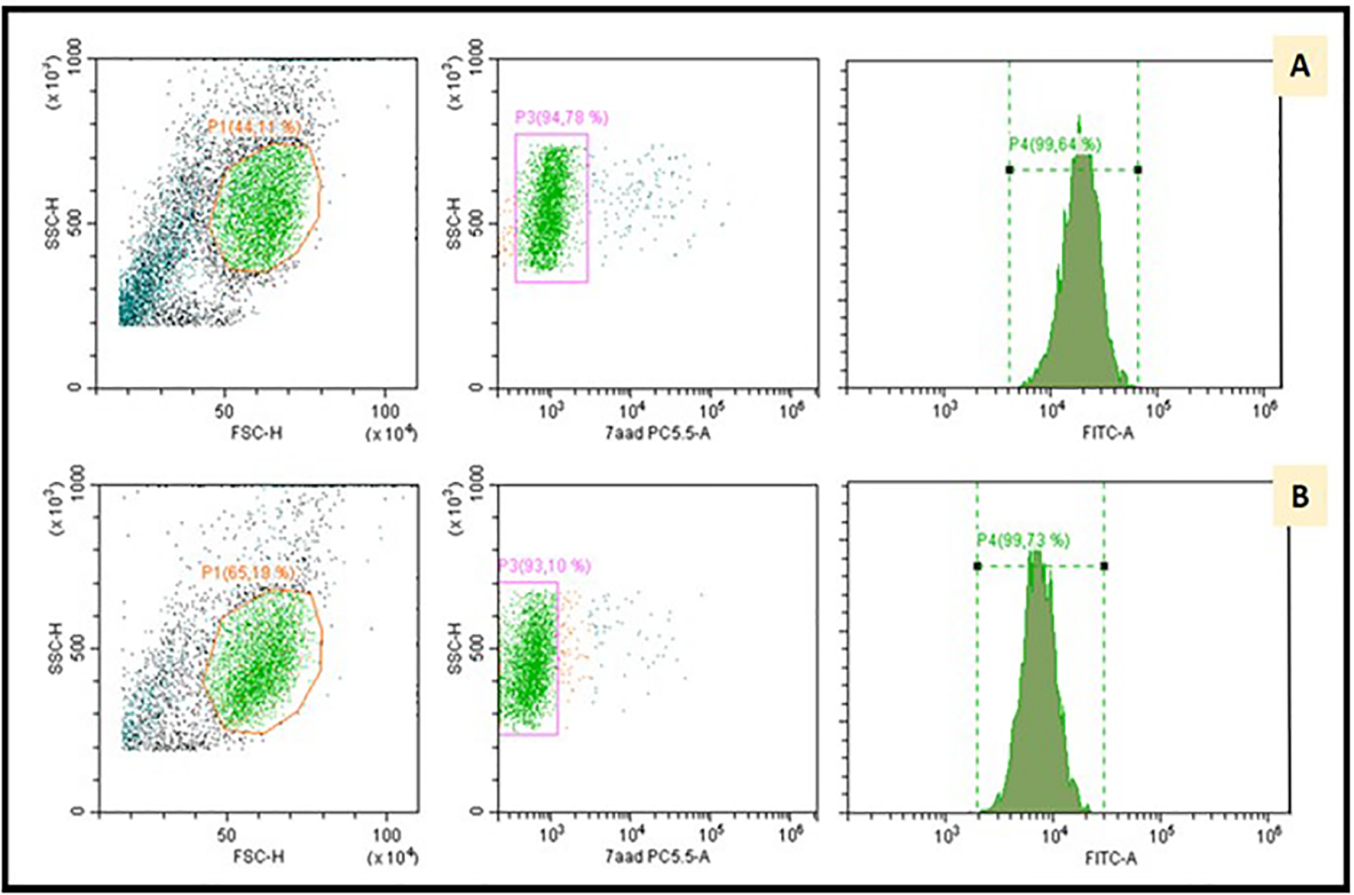
Anti-neutrophil antibody test performed by immunofluorescence technique and flow cytometry reading. The patient's granulocytes were isolated and next incubated with polyvalent IgG+IgM antiglobulin (ATG) conjugated with fluorescein isothiocyanato (FITC). To detect the presence of antibodies bound to the granulocyte membrane, live (7-aminoactinomycin D negative) neutrophils were selected and the intensity of the FITC fluorescence was analyzed. **(A)** shows our patient’s positive result. **(B)** illustrates a negative control. The study was completed after investigating the presence of free autoantibodies in the serum of the patient. In this indirect test, the patient's serum was incubated with donor granulocytes and ATG. Median fluorescence intensity was again analyzed by flow cytometry.

Bone marrow biopsy showed the absence of granulocytic lineage with normal erythroid and megakaryocytic lineages (**Figure 1**). No morphological or immunophenotypic evidence of medullary progression of WM was detected. No evidence of bone marrow dysplasia was observed. A blood immunophenotypic study of T lymphocytes, performed by flow cytometry, was normal (**Figure 2**). The diagnosis of agranulocytosis was made. Anti-neutrophil antibody test performed by immunofluorescence technique and flow cytometry reading confirmed the presence of antibodies bound to the patient’s granulocytes. However, no free anti-neutrophil antibodies were detected in the serum (**Figure 3**). The genotyped of Immunoglobulin G Fc receptor FcγRIIIa 158 polymorphic position, demonstrated the presence of the 158 V/F polymorphism, in heterozygosis.

**Figure 4:**
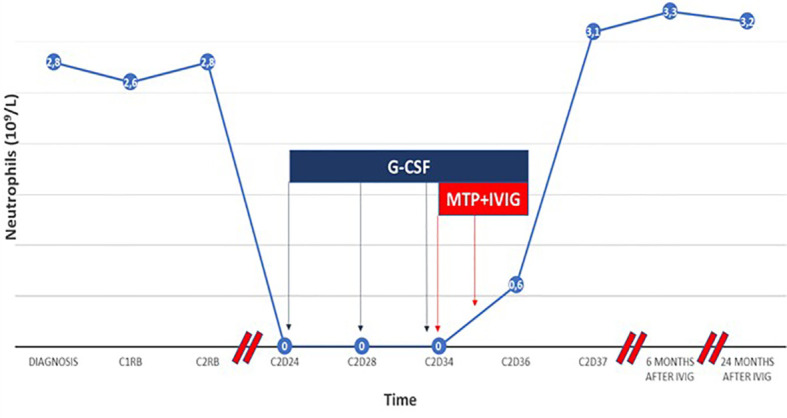
Timeline of the evolution of the patient’s neutrophil count from the diagnosis of Waldenström macroglobulinemia to the present. After 10 days of treatment with G-CSF, the patient maintained neutrophil count of 0 × 10^9^/L. The initiation of MTP and IVIG achieved a fast and lasting recovery that was established in the next 48-72h. Abbreviations: CI, first cycle; C2, second cycle;D, day within the cycle from the start of treatment; RB:Rituximab-Bendamustine.

Given the absence of response to G-CSF, treatment with methylprednisolone (MTP) 1 mg per kilogram of body weight (mg/kg bw) per day and IVIG 1 g/kg bw for two days were started. The absolute neutrophil count 48 and 72 hours later was 0.6 x10^9^/L and 3.1 x10^9^/L, respectively. The patient achieved normalization of blood counts and decreased the monoclonal component after RB, fulfilling the criteria of partial response. Full dose steroid therapy was maintained for a total of 10 days. Subsequently, a slow tapering of the prednisone dose was started until it was stopped 4 months later. The neutrophil count remained normal during the prednisone tapering dose and after it was stopped. At the last follow-up, 24 months after the agranulocytosis event and 20 months after steroid withdrawal, the patient remained in partial response and no additional episodes of neutropenia were observed (**Figure 4**).

## Discussion

The combination of R and B is widely used in non-Hodgkin lymphomas (NHL) (5). LON incidence related to R has been widely reported (3, 4, 6, 7); however, there is scarce data available regarding LON and RB regimens. Recently, Verriere B et al. described an incidence of 7% of grade III/IV LON in a series of 145 patients with chronic lymphocytic leukemia and NHL treated with RB (8). Beak et al. compared the incidence of late-onset complications in NHL patients treated with anti CD20 plus B vs anti CD20 plus CHOP (cyclophosphamide, doxorubicin, vincristine, and prednisone) or CVP (cyclophosphamide, vincristine, and prednisone). LON was significantly more common with B (10.9%) compared to CHOP/CVP (2.2%). B was also associated with a significantly increased time to neutrophil recovery (7 months with B vs 6 months with CHOP/CVP). These studies suggest that the addition of B chemotherapy to R could add long‐lasting hematological toxicity compared with other regimens (9).

The pathophysiology of LON is unknown; meanwhile, several studies have investigated the potential mechanisms in relation to R. A possible mechanism that has been reported previously is immunological disturbance due to an aberrant B-cell reconstitution and formation of autoantibodies binding to the neutrophils or its precursors (6, 7). Considering the immune-mediated mechanism presented in this case, the literature shows a perplexing overlap between the different immune neutropenia syndromes, and it is sometimes difficult to make a clear distinction between autoimmune neutropenia (AIN), pure white blood cell aplasia (PWCA), or LON (10–13). Also, a T-cell large granular lymphocyte population mediating granulocytic toxicity has been postulated due to studies that show proliferation of these cells in the bone marrow of patients with LON (14). Other studies described that B lymphocyte depletion could induce variations in growth factors, including stromal-derived factor 1 and B cell activating factor, thereby altering the normal balance between granulopoiesis and lymphopoiesis (15). In accordance with this hypothesis, a more pronounced B-lymphocyte depletion in relation to R treatment has been reported in patients with the Immunoglobulin G Fc receptor FcγRIIIa 158 V/V and V/F polymorphism (16, 17). This V/V and V/F polymorphism have been correlated with higher rates of LON in a series of patients with lymphoma treated with R compared with the FcγRIIIa 158 F/F polymorphism (18–20). We were able to demonstrate the presence of the FcγRIIIa 158 V/F polymorphism in our patient, which is consistent with an increased risk of R-induced LON development (21). Recent studies have also described maturation arrest at the (pro)myelocyte stage in patients with LON, with selective suppression of myelopoiesis (3, 22). There are no studies that specifically investigate the pathophysiology of B-induced LON. We know that B is a powerful lymphodeplective agent. Perhaps, patients treated with B have a greater disbalance between lymphopoiesis and granulopoiesis, which could confer a higher risk of developing LON. We suggest, based on our experience with this case, that the antibody-mediated immune mechanism should be considered in future research on B-induced LON pathophysiology.

Most cases of R-induced LON are self-limiting and resolve without any complications (23, 24). Although R-induced LON has the potential to be a long-lasting complication, neutrophil recovery with the use of G-CSF usually occurs in as few as four days (4, 8, 25). In the Verriere et al. series, it was not observed an increased infection rate related to LON, and the majority of the patients recover without stimulating agents. Only in some cases was G-CSF administered, and in all of them, a rapid recovery of the neutrophil count was achieved (8). The median time to onset of R-induced LON is extremely variable among series, ranging from 38 to 175 days. The median duration of LON reported ranges from 4 days up to 349 days (3, 4, 8, 26, 27). In our case, LON was detected 24 days from the last R dose and recovery (neutrophils> 1 x10^9^/L) occurred after 13 days (**Figure 4**).

Agranulocytosis associated with R unresponsive to G-CSF, as in the case reported here, is extremely rare. Treatment in this scenario is controversial. Rose et al. (28) reported one case of diffuse large B cell lymphoma that developed LON following autologous stem cell transplantation and responded to cyclosporine. Saikia et al. (29) also described a case of G-CSF resistant LON in a patient with follicular lymphoma after R maintenance therapy who responded to IVIG. G-CSF resistant R-induced agranulocytosis has also been described in association with parvovirus infections. In this specific scenario, IVIG has also been successfully used (30, 31).

## Conclusions

We present a rare case of late onset autoimmune antibody-mediated agranulocytosis in relation to RB that was resistant to G-CSF, which was successfully managed with high doses of IVIG. RB is widely used in NHL and we have to be aware of early-onset and late-onset complications that may occur related to this regimen. Patients treated with RB can develop LON, which in rare cases, can be severe and life-threatening. Whenever a severe LON after RB appears, we have to think of the possibility of agranulocytosis, especially in the G-CSF unresponsive cases. In this scenario, it must be considered that the pathogenic mechanism could be immune, and in this situation, treatment with high doses of IVIG can be quickly effective.

## Data Availability Statement

The original contributions presented in the study are included in the article/supplementary material, further inquiries can be directed to the corresponding author/s.

## Author Contributions

RD-F, JR-S, and AS wrote the original manuscript. SF, AF, LC, and C-FR helped in the diagnosis, treatment, and evaluation section. All authors contributed to the article and approved the submitted version.

## Conflict of Interest

The authors declare that the research was conducted in the absence of any commercial or financial relationships that could be construed as a potential conflict of interest.

## Publisher’s Note

All claims expressed in this article are solely those of the authors and do not necessarily represent those of their affiliated organizations, or those of the publisher, the editors and the reviewers. Any product that may be evaluated in this article, or claim that may be made by its manufacturer, is not guaranteed or endorsed by the publisher.
